# Synergistic Anti-tumor Effect of Dichloroacetate and Ivermectin

**DOI:** 10.7759/cureus.21884

**Published:** 2022-02-03

**Authors:** Tatsuaki Ishiguro, Ryumei H Ishiguro, Miyu Ishiguro, Atsushi Toki, Hiroshi Terunuma

**Affiliations:** 1 Tumor Biology, Kamui Medical Co. Ltd., Tokyo, JPN; 2 Internal Medicine, Tama Nanbu Chiiki Hospital, Tokyo, JPN; 3 Internal Medicine, N2 Clinic Yotsuya, Tokyo, JPN

**Keywords:** sarcoma, cancer, tamoxifen, omeprazole, ivermectin, dichloroacetate

## Abstract

We formerly reported that the combination of dichloroacetate, omeprazole, and tamoxifen blocked cancer progression by reducing lactic acid production and inducing superoxide production. Recently, ivermectin, a well-known anti-parasite drug, was reported to share the same mechanisms with them and have anti-tumor activity. Here, we present three patients in whom the combination of dichloroacetate, omeprazole (plus tamoxifen), and ivermectin dramatically relieved the symptoms accompanying cancer and sarcoma progression.

## Introduction

An increasing number of clinically approved non-cancer drugs have been proved to have antitumor activity. Among those was dichloroacetate, an approved drug for congenital lactic acidosis [1]. Warburg discovered that even in the presence of sufficient oxygen, cancer cells metabolize glucose and produce lactic acid [2-4]. Bonnet et al. reported that dichloroacetate reverses the Warburg effect and inhibits tumor growth [5]. Dichloroacetate increases the flux of pyruvate into the mitochondria by inhibiting pyruvate dehydrogenase kinase and promotes glucose oxidation over glycolysis. As a result, dichloroacetate decreases the production of lactic acid and increases superoxide production, which relieves the symptoms accompanying cancer progression and induces mitochondria-dependent apoptosis.

We formerly reported that omeprazole (a proton pump inhibitor) enhances the antitumor effect of dichloroacetate by the alkalinization of lysosomes and the permeabilization of lysosome membranes followed by the production of reactive oxygen species [6-7]. Tamoxifen also induces caspase-dependent cancer cell growth inhibition through superoxide production and is used not only for hormone receptor-positive breast cancer but also for pancreatic cancer and sarcoma [8-9]. Taken together, we showed that the addition of tamoxifen to the combination of dichloroacetate and omeprazole strengthens their effects on fibrosarcoma cells in vitro [10]. This combination also blocked cholangiocarcinoma progression for three months [10].

On the other hand, ivermectin, an avermectin derivative used to treat parasites, was reported to have a growth inhibitory effect on cancer cells through the induction of mitochondrial dysfunction and oxidative damage [11-12]. Since ivermectin shares the same anti-tumor mechanism as dichloroacetate, omeprazole, and tamoxifen, we postulated that a full or partial combination of these drugs might be a promising protocol for treating malignant tumors.

## Case presentation

Here, we show three cases successfully treated with a full or partial combination of dichloroacetate, omeprazole (plus tamoxifen), and ivermectin.

Case 1

**Figure 1:**
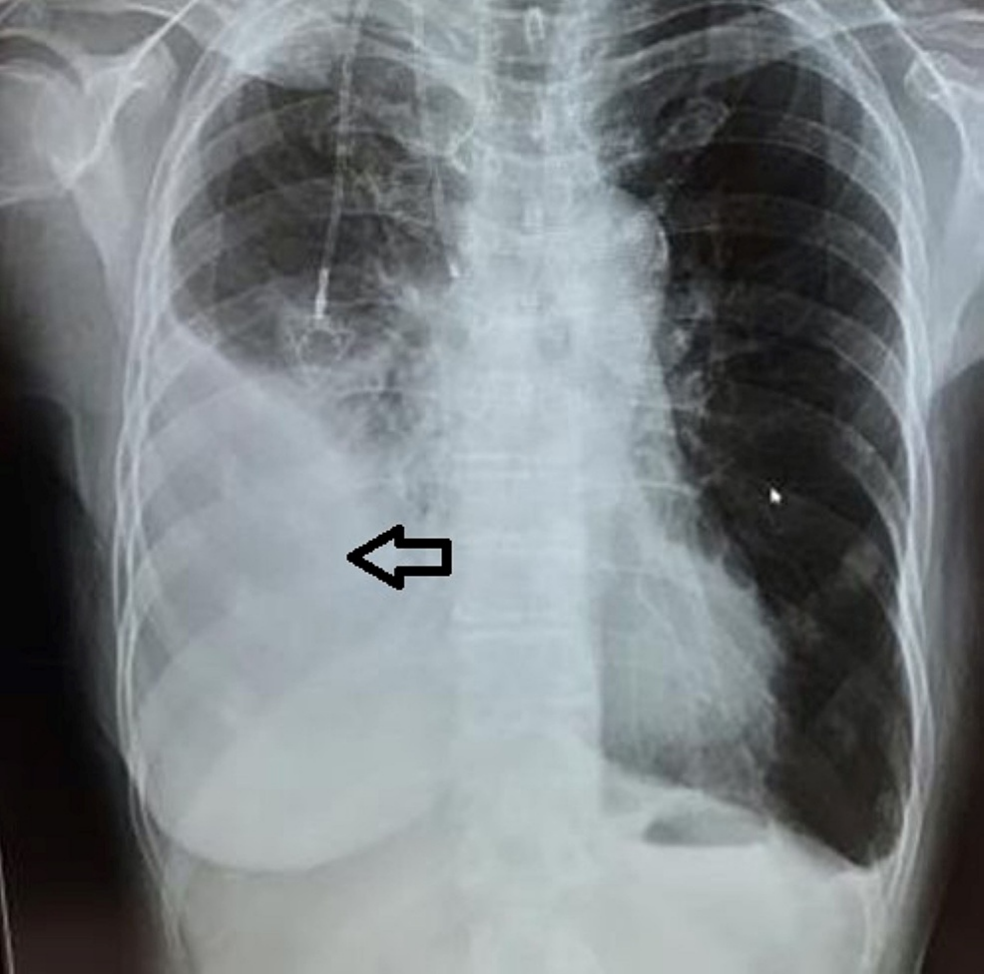
CXR showing pleural effusion due to breast cancer dissemination CXR: chest X-ray

A 69-year-old female was diagnosed with invasive breast cancer in October 2015. She underwent left breast partial mastectomy, but bone metastases and pleural dissemination appeared (Figure 1). She responded to chemo-endocrine therapy, but it soon became less effective, and intolerable side effects appeared. We gave up conventional therapy and started treatment with dichloroacetate, omeprazole, and tamoxifen from October 2021: weekly use of three tablets of 333 mg dichloroacetate per day (on Day 1), three tablets of 40 mg omeprazole per day (on Day 1), along with a tablet of 20 mg tamoxifen every day. This relieved her symptoms (bone pain, shortness of breath, and general fatigue) instantly but not completely. Hence, we added a tablet of 12 mg ivermectin per day (on Day 1), which stabilized pleural effusion and induced tumor marker reduction (CEA or carcinoembryonic antigen), which went down from 12.9 to 7.3, and cancer antigen 15-3 (CA15-3), which went down from 302.3 to 229.4 in three months).

Case 2

**Figure 2:**
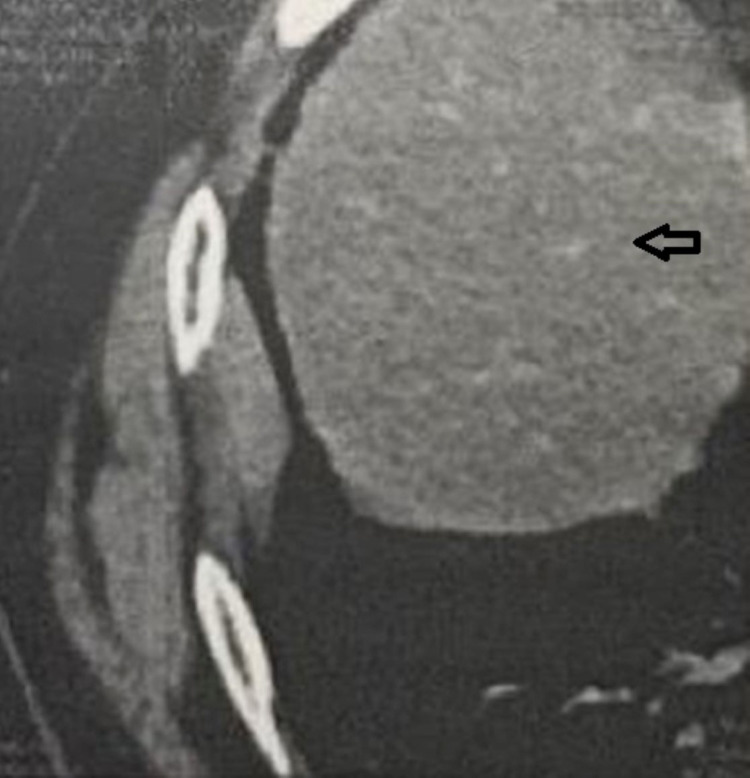
CT showing pleural metastasis of osteosarcoma

A 54-year-old male was diagnosed with right femur osteosarcoma in February 2021. He underwent neoadjuvant chemotherapy with adriamycin and ifosfamide followed by tumor resection but local recurrence occurred. Although he received chemotherapy with ifosfamide, doxorubicin, and cisplatin, lung lymph node metastases and pleural dissemination appeared (Figure 2). He could not walk out of his house himself because of shortness of breath and severe pain. Since the combination of dichloroacetate, omeprazole, and tamoxifen had not been effective in sarcoma patients in our study, we decided to add ivermectin to them: weekly use of three tables of 333 mg dichloroacetate per day (on Days 1 and 4), three tablets of 40 mg omeprazole tablets per day (on Days 1 and 4), a tablet of 20 mg tamoxifen per day (on Days 1 and 4), along with a tablet of 12 mg ivermectin per day (on Days 1 and 4). After only one cycle, all the symptoms were relieved dramatically, and he could come to our clinic on foot by himself.

Case 3

**Figure 3:**
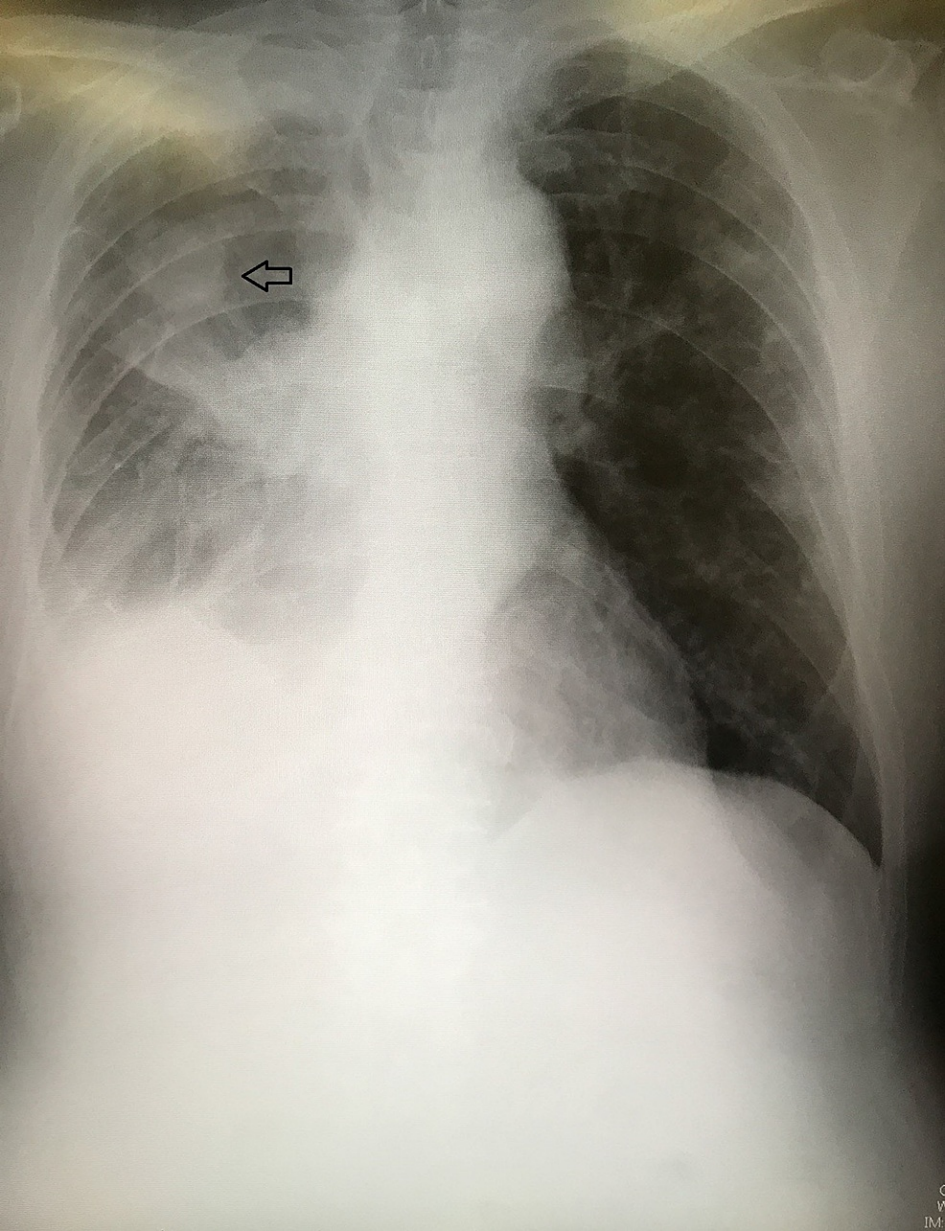
CXR showing the right lung adenocarcinoma and pleural effusion due to dissemination CXR: chest X-ray

A 54-year-old male was diagnosed with right lung adenocarcinoma in September 2014 (Figure 3) and underwent right lung lobectomy. Although he underwent postoperative chemotherapy, local recurrence with lymph node metastases appeared in one year. Initially, chemotherapy was effective, but gradually, it became less effective and metastases in other sites (bone and brain) appeared. Even after we started the combination therapy with a tablet of 333 mg dichloroacetate every two days, a tablet of 40 mg omeprazole every two days, and a tablet of 12 mg ivermectin per week, his symptoms (cough, shortness of breath, pain, and appetite loss) did not improve. However, soon after we changed our protocol (a tablet of 12 mg ivermectin twice a week), all the symptoms were relieved.

## Discussion

Cancer-associated pain is a major cause of increased morbidity and diminishes the quality of life. Cancer cells release protons and lactate via plasma membrane pH regulators to avoid the intracellular acidification resulting from increased aerobic glycolysis, known as the Warburg effect [2-3]. Since acidosis is algogenic for sensory neurons, the acidic microenvironments can evoke cancer-associated pain.

Dichloroacetate inhibits the Warburg effect. It inhibits pyruvate dehydrogenase kinase, which blocks pyruvate dehydrogenase through its phosphorylation activity [5]. When pyruvate dehydrogenase kinase is inhibited by dichloroacetate, pyruvate dehydrogenase is reactivated, causing the mitochondria to no longer be hyperpolarized; instead, the membrane and mitochondria are depolarized, reactivating the mitochondrial K+ channels, which then decrease cytosolic K+. When pyruvate dehydrogenase is inhibited in cancer cells by pyruvate dehydrogenase kinase, an excess cytosolic K+ occurs that inactivates caspases 3 and 9. Dichloroacetate reactivates these caspases along with an increase in hydrogen peroxide intracellularly, allowing the release of cytochrome c from the mitochondria. The release of cytochrome c is a major activating step for cell apoptosis, as it triggers the caspase cascade.

On the other hand, the postulated mechanisms of the antitumor effects of ivermectin are: 1) inhibition of the multidrug resistance protein P-glycoprotein, 2) osmotic cancer cell death by the upregulation of the chloride channel, 3) P2X4/P2X7- and caspase-1-mediated immunogenic cancer cell death, 4) inhibition of the PAK1/Akt/mTOR pathway, 5) inhibition of the WNT-TCF pathway, 6) blockade of the PAH2-SID interaction, 7) inhibition of the helicase activity, 8) reduction of cancer stem cell populations accompanying the downregulation of Nanog, Oct4, and Sox2, 9) induction of anti-angiogenic effects, and 10) mitochondrial dysfunction with oxidative damage [13].

Proton pumps like the vacuolar-type H+ ATPase (V-ATPase) are involved in controlling cellular pH in normal and tumor cells. Treatment with proton pump inhibitors induces the sensitization of cancer cells to chemotherapeutics via modifications of cellular pH gradients. This effect of proton pump inhibitors is mediated by the very early production of reactive oxygen species. Tamoxifen also induces reactive oxygen species-mediated autophagic cell death [10].

Dichloroacetate, omeprazole, tamoxifen, and ivermectin share the same anti-tumor mechanism of mitochondrial dysfunction followed by reactive superoxide production. The full or partial combination of them was expected to reduce the necessary dose of each drug and the accompanying risks. Although dichloroacetate alone was reported to reduce severe cancer pain and rarely eradicate tumors [14-15], most chemotherapy-resistant cancer and sarcoma have not responded to the combination of dichloroacetate, omeprazole, and tamoxifen in our study [10]. However, in this report, we presented three cases in which the addition of ivermectin to the combination of dichloroacetate and omeprazole (plus tamoxifen) was effective. Although our rapid communication is preliminary, we think our protocol may open new therapeutic options.

## Conclusions

We formerly reported that omeprazole and tamoxifen enhanced the antitumor effect of dichloroacetate by the production of reactive oxygen species, and this combination blocked cholangiocarcinoma progression for three months. Ivermectin, an avermectin derivative used to treat parasites, was reported to have a growth inhibitory effect on cancer cells through the induction of mitochondrial dysfunction and oxidative damage. Since all these drugs share the same anti-tumor mechanism, we postulated that a full or partial combination of these drugs might be a promising protocol, and in this report, we showed that combination therapy with dichloroacetate, omeprazole (plus tamoxifen), and ivermectin was extremely effective to relieve the symptoms accompanying cancer and sarcoma.
